# Incidence, subtypes, sex differences and trends of stroke in Taiwan

**DOI:** 10.1371/journal.pone.0277296

**Published:** 2022-11-16

**Authors:** Chung-Fen Tsai, Ya-Hui Wang, Nai-Chi Teng, Ping-Keung Yip, Li-Kwang Chen

**Affiliations:** 1 Division of Neurology, Cardinal Tien Hospital and School of Medicine, Fu Jen Catholic University, New Taipei City, Taiwan; 2 Medical Research Center, Cardinal Tien Hospital and School of Medicine, Fu Jen Catholic University, New Taipei City, Taiwan; 3 Institute of Population Health Sciences, National Health Research Institutes, New Taipei City, Taiwan; Federal Medical Centre Umuahia, NIGERIA

## Abstract

**Background:**

Chinese populations have been reported higher incidence of all strokes and intracerebral hemorrhage. However, few large-scale studies have evaluated changes of stroke epidemiology in the 21st century.

**Methods:**

We explored the rates of incidence of all first-ever strokes, subtypes, and 1-month case fatality by using data from the Taiwan National Health Insurance Research Database since 2004. Also, we investigated sex differences in stroke. Time-trend analysis was performed for incidence and case fatality rates of all strokes and subtypes in both sexes.

**Results:**

The age-adjusted incidence of all strokes per 100,000 person-years decreased by 16%, from 251 (95% confidence interval [CI] 249–253) in 2004 to 210 (95% CI 209–212) in 2011 (p<0.001); it was always higher in Chinese men than in women. Among pathological subtypes, the incidence of intracerebral hemorrhage markedly decreased by 26% over the years (p<0.001), while that of ischemic stroke slightly decreased by 8%. However, when stratified by sex, the incidence of ischemic stroke decreased significantly in only women, not in men (men: p = 0.399, women: p = 0.004). Regarding the incidence of subarachnoid hemorrhage, it remained unchanged. Furthermore, the rate of 1-month case fatality decreased significantly for all strokes in both sexes (p<0.001).

**Conclusions:**

In Taiwan, the incidence rate of first-ever stroke decreased in both Chinese men and women in the early 21st century. Men had a higher incidence rate than women. Furthermore, a marked decrease was noted in the incidence of intracerebral hemorrhage, while a slight decrease was noted in that of ischemic stroke; however, the decreased incidence of ischemic stroke was significant in only women.

## Introduction

Stroke is a major global disease and a major public health challenge and is the second leading cause of death. Although stroke mortality and incidence rates have decreased in many countries in recent decades, the absolute number of deaths and disabled people due to stroke has increased substantially, leading to heavy burdens in the world [1].

The incidence and mortality rates, and subtypes of stroke vary across regions, populations, and biological sexes [2]. Earlier studies reported that stroke was more common in men than in women, but women had more severe stroke, with higher 1-month case fatality rates than those of men [2, 3]. However, most studies have been conducted in Western populations. While age-adjusted incidence of stroke has decreased in many Western countries in the last two decades, it has increased significantly in East Asia, especially in China [4]. Compared with western populations, Chinese populations have higher incidence of stroke, younger age of onset, and a twofold higher proportion of intracerebral hemorrhage (ICH) [5, 6]. Nevertheless, in the 21st century, few large-scale population-based studies have been conducted to investigate the changes of incidence rates of stroke, subtypes, and early case-fatality rates in Chinese populations in Taiwan. Moreover, the possible changes in sex differences require further investigation.

For prevention of stroke to be effective in Chinese populations, it is important to have more reliable and accurate stroke-related data. To test the hypothesis that the incidence and 1-month case fatality rates, subtypes, and trends of stroke would have changed in the 21st century and would vary between Chinese men and women, we conducted this study by using data from Taiwan National Health Insurance Research Database (NHIRD). A good understanding of stroke epidemiology could facilitate better prediction of the potential impacts influenced by aging populations and changing lifestyle, thus developing more effective strategies for stroke prevention.

## Methods

### Study design and population

We conducted a nationwide, retrospective cohort study by using prospectively recruited data from Taiwan NHIRD. Taiwan government has implemented the compulsory National Health Insurance (NHI) program since 1995, including data on the ambulatory, outpatient clinics and inpatient records, medical examinations, and prescriptions of approximately 99% of the total 23 million people in Taiwan. All data from NHI program were transformed into NHIRD to have comprehensive records of medical care. In the present study, we used random sampling of 2 million patients from 2004. All data were fully anonymized before we accessed them. The Ethics Committee of Cardinal Tien Hospital had waived the requirement for informed consent and approved this study.

### Inclusion of stroke and classification of subtypes

We included first-ever acute Chinese stroke patients of any age from hospitals and outpatient clinics. Acute stroke was defined as a new diagnosis of stroke, and pathological subtypes were classified according to the following codes of the International Classification of Diseases, Ninth Revision (ICD-9)—subarachnoid hemorrhage (SAH: 430), intracerebral hemorrhage (ICH: 431), ischemic stroke (IS: 433.01, 433.11, 433.21, 433.31, 433.81, 433.91, 434.01, 434.11, and 434.91), and unclassified type (436) during study period [7, 8].

The enrolled patients had undergone brain computed tomography (CT) or magnetic resonance imaging (MRI) within one month of the new diagnosis of stroke. We excluded patients with any type of stroke before the study, traumatic brain hemorrhage, subdural/epidural hemorrhage, brain tumor or inflammation, or transient ischemic attack.

### Statistical analysis

We calculated crude as well as age- and sex-specific incidence of all first-ever strokes and subtypes per 100,000 person-years with 95% confidence intervals (CIs) by using Poisson distribution for the whole population per year from 2004 to 2011. The denominator for the incidence rate was based on the census data of Ministry of the Interior, Taiwan. Incidence was standardized to World Health Organization (WHO) World Standard population. The age-specific incidence of stroke and its subtypes were investigated for the following age bands for all strokes: <15, 15–24, 25–34, 35–44, 45–54, 55–64, 65–74, 75–84, >85 years, without limitation of age or hospitalization. To compare the differences in incidence between male and female patients, we calculated sex-specific incidence rates over the years. Also, we conducted linear trend analyses for all strokes and subtypes by using Poisson regression model, with year as the continuous variable. In addition, we calculated Incidence Rate Ratio (IRR) for 2011 versus 2004, as well as male-to-female incidence ratios per year.

For 1-month case fatality rate, we calculated the percentage of acute stroke patients who died within 1 month based on death certificate record, and we used Cochran-Armitage trend test to investigate the temporal changes over the years for all strokes and subtypes. All statistical tests were two-sided, and *p* values less than 0.05 were considered significant. Statistical analyses were performed with SAS version 9.4 (SAS Institute Inc, Cary, NC, USA).

## Results

### Incidence and trends of all first-ever strokes

**Fig 1:**
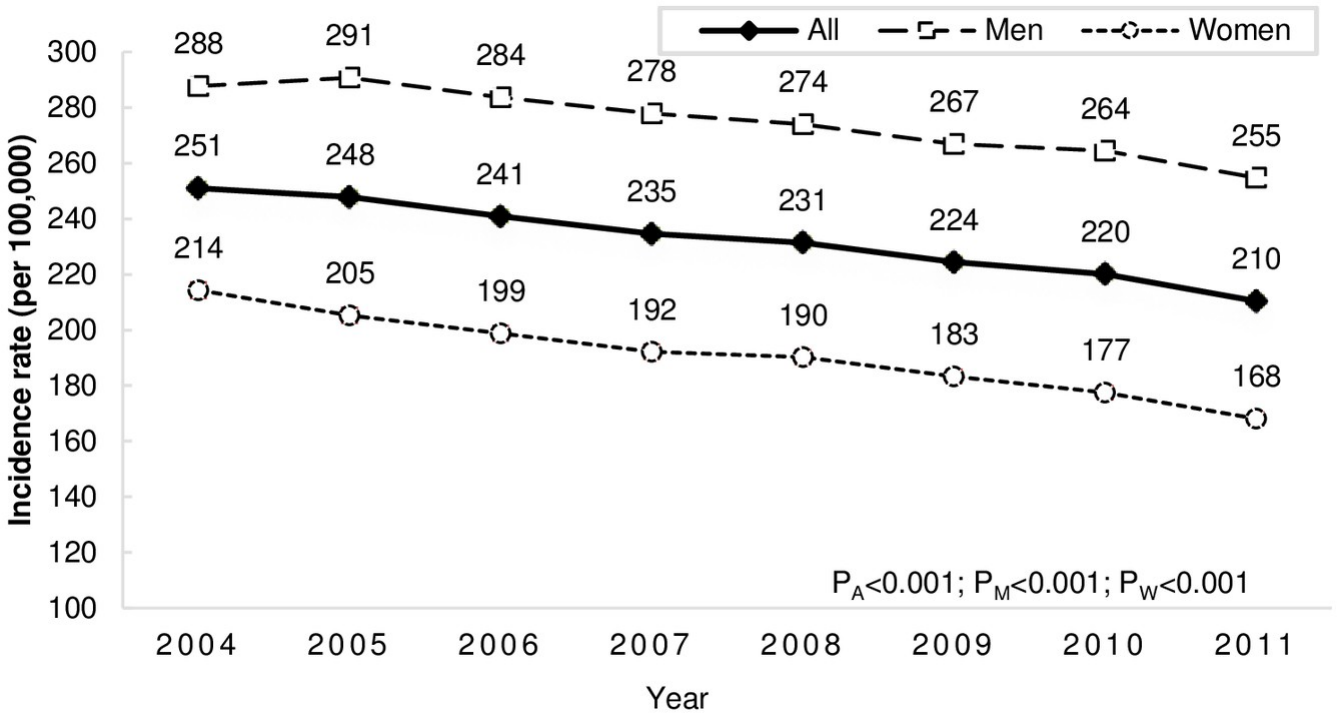
Trends of age-adjusted incidence of all first-ever strokes. P_All_ = p for trend in all strokes; P_M_ = p for trend in men; P_W_ = p for trend in women.

During the study period, we identified a total of 371,846 (weighted number = 523,726) first-ever stroke patients, 58% were men. Of them, 73% were admitted to hospitals. The mean age of stroke onset was 66.6 years for all strokes; the age of onset was lower in men than in women (men: 65.1 years, women: 68.5 years, p<0.001). Table 1 summarized the age- and sex-specific adjusted incidence of all first-ever strokes. As expected, the annual incidence increased with increasing age and was higher in men than in women every year, except in patients aged ≥ 85 years. The age-adjusted incidence of all strokes decreased by 16% (IRR 0.84 for 2011 versus 2004, 95% CI 0.83–0.85), from 251 (95% CI 249–353) per 100,000 person-years in 2004 to 210 (95% CI 209–212) in 2011 (Fig 1). The decrease of incidence was more in women—21% decrease for 2011 versus 2004 (IRR 0.79, 95% CI 0.77–0.80), while less in men—only 11% decrease during the same period (IRR 0.89, 95% CI 0.88–0.90). The flow chart of inclusion of acute stroke patients was shown in S1 Fig.

**Table 1:** Age and sex-specific adjusted incidence of all first-ever strokes (per 100,000 person-years) in Chinese populations in Taiwan.

Year	2004	2005	2006	2007	2008	2009	2010	2011
**Age/N**	62884	64252	64841	65465	66610	66520	67193	65962
**<15**	6 (6–7)	7 (6–8)	7 (7–8)	6 (5–7)	6 (6–7)	7 (6–7)	7 (6–7)	6 (5–7)
**15–24**	22 (21–24)	25 (24–27)	23 (22–25)	22 (20–23)	22 (21–24)	22 (20–23)	22 (21–24)	22 (20–24)
**25–34**	33 (31–35)	34 (33–36)	34 (32–36)	35 (33–37)	33 (32–35)	38 (36–40)	36 (35–38)	36 (34–38)
**35–44**	87 (84–90)	86 (83–89)	85 (82–88)	89 (86–93)	90 (87–93)	90 (87–93)	90 (87–93)	88 (85–91)
**45–54**	262 (257–268)	248 (243–253)	249 (244–254)	243 (238–248)	244 (238–249)	238 (233–243)	230 (225–235)	219 (214–223)
**55–64**	673 (661–685)	640 (629–652)	617 (606–629)	577 (566–587)	562 (552–572)	551 (541–560)	514 (505–523)	492 (484–500)
**65–74**	1387 (1367–1408)	1378 (1358–1398)	1318 (1298–1337)	1276 (1257–1295)	1249 (1230–1267)	1181 (1163–1199)	1168 (1151–1186)	1110 (1092–1127)
**75–84**	2215 (2181–2250)	2243 (2209–2277)	2210 (2177–2243)	2177 (2145–2210)	2147 (2116–2179)	2063 (2033–2094)	2081 (2051–2111)	1993 (1964–2023)
**85+**	2767 (2682–2853)	2773 (2691–2856)	2667 (2590–2744)	2739 (2664–2813)	2774 (2701–2846)	2679 (2610–2748)	2702 (2636–2768)	2553 (2492–2615)
**AIR for all**	251 (249–253)	248 (246–250)	241 (239–243)	235 (233–236)	231 (230–233)	224 (223–226)	220 (218–222)	210 (209–212)
**AIR for M**	288 (285–291)	291 (288–294)	284 (281–287)	278 (275–281)	274 (271–277)	267 (264–270)	264 (262–267)	255 (252–257)
**AIR for W**	214 (212–217)	205 (203–208)	199 (196–201)	192 (190–195)	190 (188–192)	183 (181–185)	177 (175–180)	168 (166–170)

N = number of first-ever strokes; AIR = adjusted incidence rate; M = men; W = women.

### Incidence and trends among pathological types of strokes

**Fig 2:**
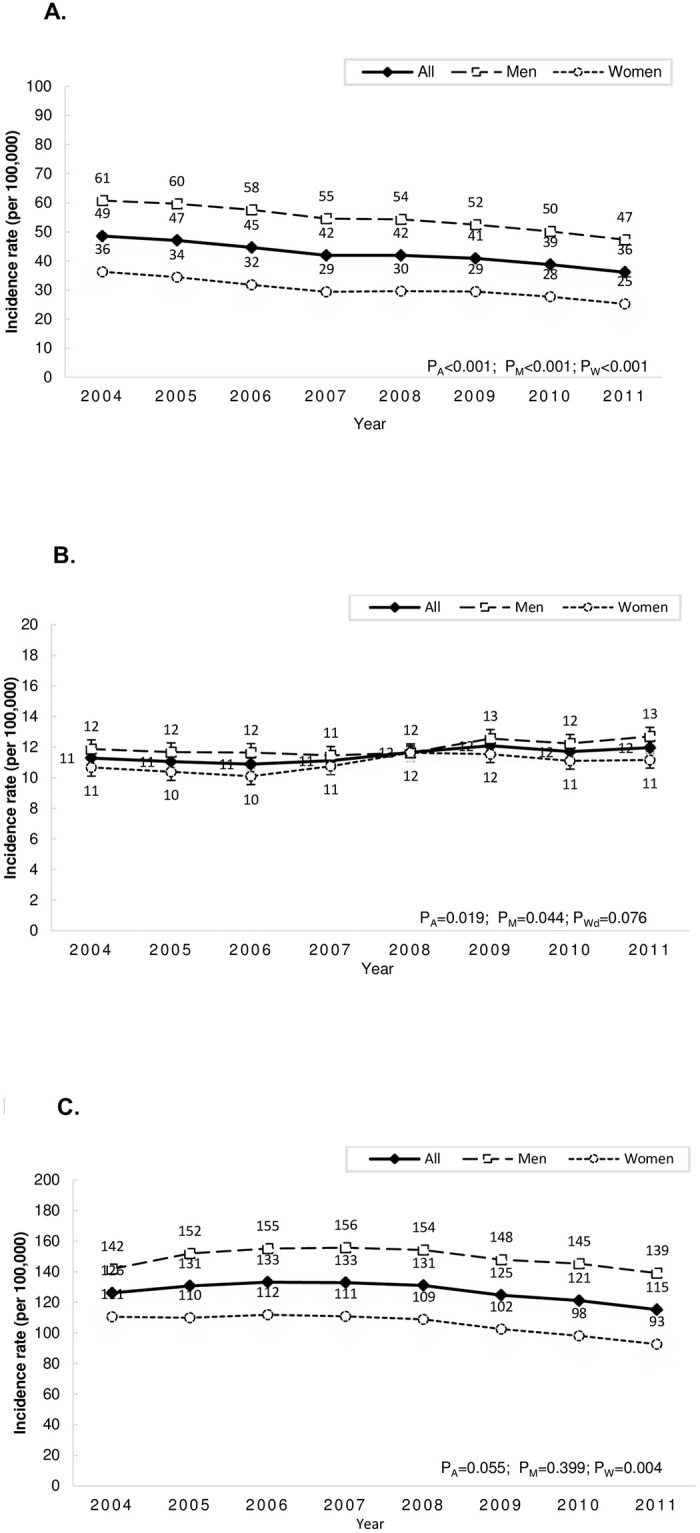
**A**. Trends of incidence of intracerebral hemorrhage. **B**. Trends of incidence of subarachnoid hemorrhage. **C**. Trends of incidence of ischemic stroke. P_All_ = p for trend in all strokes; P_M_ = p for trend in men; P_W_ = p for trend in women.

Among all patients with first-ever strokes, 18% had ICH and most of them were men (men 64%, women 36%). The mean age of ICH onset was 59.9 years, and male ICH patients were younger at onset than female patients (men 58.3 years, women 62.8 years, p<0.001). The age-adjusted incidence of ICH decreased year by year from 49 per 100,000 person-years (95% CI 48–49) in 2004 to 36 (95% CI 35–37) in 2011 (Fig 2A); it was always much higher in men than in women. Furthermore, the adjusted incidence of ICH markedly decreased by approximately 26% (IRR 0.74, 95% CI 0.73–0.77) in all, 22% in men (IRR 0.78, 95% CI 0.76–0.81), and 31% in women (IRR 0.69, 95% CI 0.66–0.72). As for SAH, it accounted for 5% of all strokes (men, 51%). The mean age of onset was 54.4 year; patients with SAH were younger than those with other subtypes (men 51.8 years, women 57.8 years, p<0.001). The age-adjusted incidence of SAH remained almost unchanged between 2004 and 2011 in both men and women (IRR in all 1.05, 95 CI% 1.00–1.11; IRR in men 1.07, 95 CI% 0.99–1.14; IRR in women 1.04, 95% CI 0.96–1.11) (Fig 2B).

IS was still the most common subtype of stroke. The mean age onset was 69.3 years (men, 58%); patients with IS were older than those with other subtypes of stroke (men 68.0 years, women 71.2 years, p<0.001). The age-adjusted incidence of IS slightly decreased by approximately 8% (IRR 0.92, 95% CI 0.91–0.93) in all patients. However, when stratified by sex, the decrease in the age-adjusted incidence of IS was significant only in women (IRR 0.84, 95% CI 0.82–0.86), not in men (IRR 0.99, 95% CI 0.97–1.01) (Fig 2C).

Over the years, a shift was noted in the distribution of pathological types of stroke: a decreasing proportion of ICH and an increasing proportion of IS from 2004 to 2011. Thus, the ICH-to-IS incidence ratio decreased significantly in all patients; however, it was always higher in men than in women (S2 Fig).

### Changes in the male-to-female incidence ratio for all strokes and subtypes

**Fig 3:**
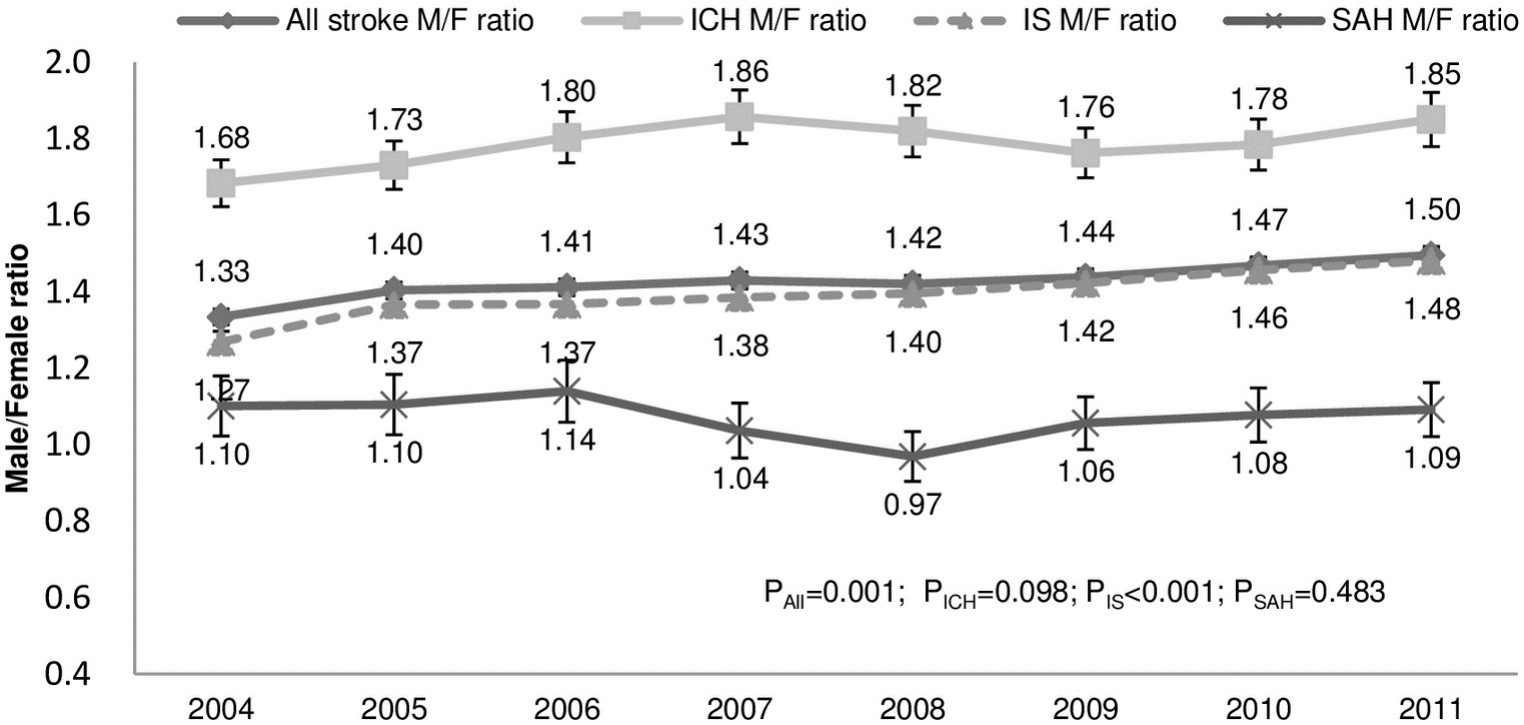
Changes in the male-to-female incidence ratio for all strokes and subtypes. M = male; F = female; ICH = intracerebral hemorrhage; IS = ischemic stroke; SAH = subarachnoid hemorrhage; P_All_ = p for trend in all strokes; P_ICH_ = p for trend in ICH; P_IS_ = p for trend in ischemic stroke; P_SAH_ = p for trend in subarachnoid hemorrhage.

In addition, we found changes of sex differences in the incidence of all strokes and IS during the study period, with an increasing male-to-female incidence ratio: all strokes, from 1.33 (95% CI 1.31–1.36) in 2004 to 1.50 (95% CI 1.47–1.52) in 2011 (p = 0.001), and IS, from 1.27 (95% CI 1.24–1.30) in 2004 to 1.48 (95% CI 1.45–1.51) in 2011 (p<0.001) (Fig 3). These changes were not significant for ICH or SAH.

### One-month case fatality for all strokes and subtypes

**Fig 4:**
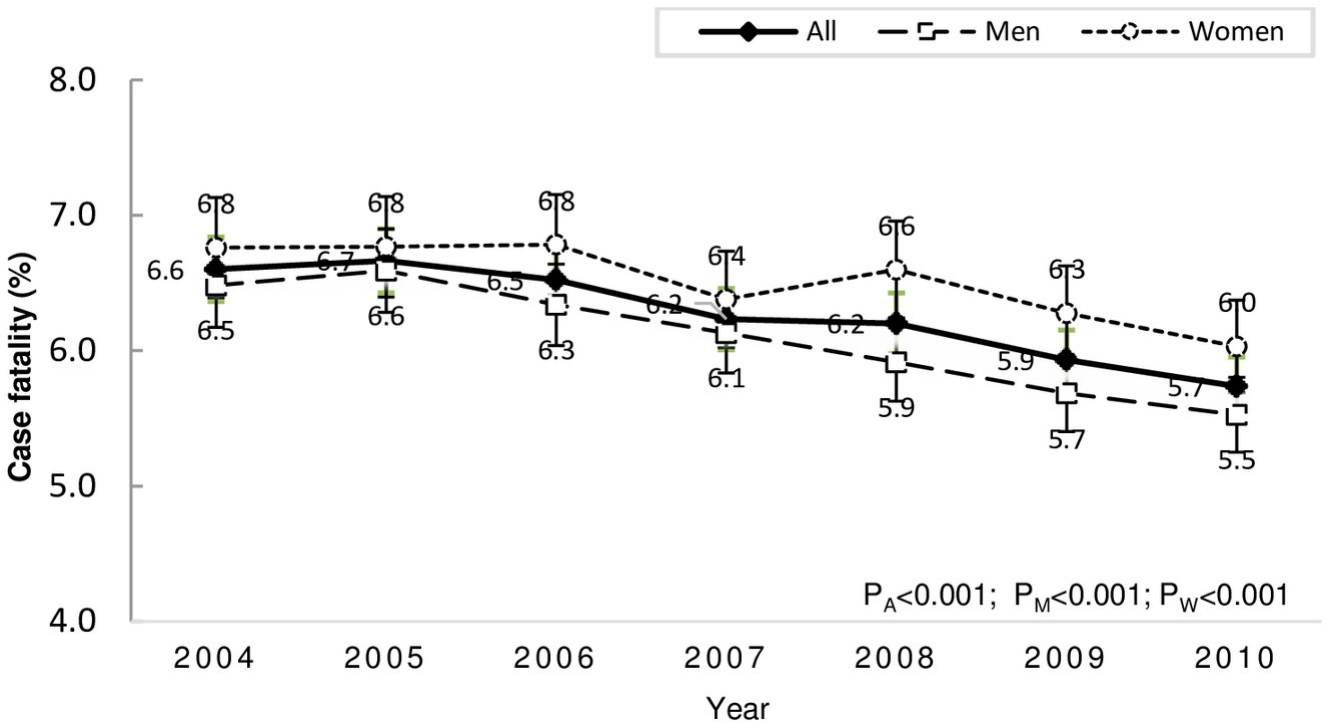
Trends of 1-month case fatality of all strokes. P_All_ = p for trend in all strokes; P_M_ = p for trend in men; P_W_ = p for trend in women.

The rate of 1-month case fatality for all strokes reduced significantly over the years, from 6.6% (95% CI 6.4–6.8%) in 2004 to 5.7% (95% CI 5.5–6.0%) in 2011, without statistical difference between men and women (Fig 4). For pathological subtypes, the decreases were significant for hemorrhagic stroke: SAH, from 18% (95% CI 16–20%) to 16% (95% CI 14–17%), and ICH, from 16% (95% CI 15–16%) to 14% (95% CI 13–14%); it remained unchanged for IS (approximately 4.0%).

## Discussion

Our study showed that the age-adjusted incidence of all strokes in Taiwan decreased annually in the early 21st century in both Chinese men and women (by 16% from 2004 to 2011). Notably, it was always higher in men than women; the trend of male-to-female incidence ratio also increased during the study period. Among pathological types of stroke, the adjusted incidence of ICH decreased markedly by 26% over the years, while the adjusted incidence of IS decreased slightly by 8% in all. Thus, the incidence ratio of ICH/IS decreased significantly over the years. However, when stratified by sex, the adjusted incidence of IS reduced significantly in only women, not in men. Regarding SAH, it remained unchanged overtime. The rate of 1-month case fatality decreased significantly for all strokes in both men and women.

Stroke is the first leading cause of deaths in China and the fourth in Taiwan, thus imposing a heavy burden on Chinese populations [6, 9]. Our systematic review has shown that Chinese have a higher incidence rate of stroke as compared with Caucasians [5]. Also, the incidence of stroke varies considerably, from north to south and urban to rural regions [5, 6, 10, 11]. In addition to geographical variations, sex differences have been reported in different populations [12–14]. An earlier systematic review from Caucasians reported that the incidence of stroke was approximately 30% higher in men than in women [3]. In our study conducted in Taiwan, the difference was even higher—Chinese men had 33–50% higher incidence of all strokes than that in Chinese women; an increasing trend was noted in male-to-female incidence ratio during the study period. This might relate to differences in risk factor prevalence, adequate treatment, education levels, socioeconomic and socio-cultural factors, genetic factors, as well as differences in sex hormones’ effects on cerebral circulation between men and women [15, 16]. More sex-specific studies are needed to clarify the disparities.

In contrast to the overall increasing incidence of stroke in China [6, 10], we demonstrated a decreasing incidence rate of all strokes in Taiwan in the 21st century. Compared with earlier studies in Taiwan before 2000, our results also showed a lower incidence rate of stroke [17, 18]. This decrease of incidence was higher in women than in men. Our findings were different from that of the Greater Cincinnati Study, which reported that the decrease of incidence of stroke over time was higher in men than in women [19]. Nevertheless, the difference in trends of stroke in our study were consistent with another study in Taiwan, which included adult hospitalized patients with IS [20]. However, some stroke patients might not have been hospitalized due to mild severity of stroke or other reasons, which might have introduced a bias. Herein, we used the Taiwan NHIRD data to investigate the incidence and trends of all first-ever strokes and subtypes in patients of all ages, including inpatients and outpatients. Therefore, it reduced the aforementioned bias of variable hospital admission rates.

In our study, the adjusted incidence decreased substantially for ICH in both sexes, while it decreased only slightly for IS. Thus, the incidence ratio of ICH/IS declined over time. Our findings were similar to those studies conducted in urban China, Japan and other high-income countries [21–23] but differed from a study conducted in rural China, which showed increasing incidence rates in both ICH and IS [24]. However, when stratified by sex, the adjusted incidence of IS decreased significantly in only women, not in men. In literature, hypertension and alcohol intake are associated with ICH, whereas diabetes, hyperlipidemia, and atrial fibrillation are associated with IS [25, 26]. Improvement of hypertensive control and less alcohol intake in Taiwan may account for the decreased incidence of ICH [27]. Nonetheless, the prevalence of diabetes, hyperlipidemia and atrial fibrillation has recently increased in Chinese populations [28, 29]. The findings in Taiwan could be useful to predict stroke epidemiology in China or other developing countries with changing economy and lifestyle, along with aging populations. Thus, improving education, better diet control, exercise and lifestyle modifications, and adequate treatment of these risk factors are important to reduce the incidence of IS in the future.

As for 1-month case fatality, our result showed a decreasing trend for all strokes over the years, without significant difference between the sexes. Compared with the findings of other studies, our results showed a lower rate of 1-month case fatality [3, 12]. The decrease in early case fatality after stroke in Taiwan may reflect the improvement of acute stroke care in recent years, including more thrombolytic treatment, better management of severe strokes, higher detection of minor strokes using MRI, and affordable expenses under the NHI program in Taiwan [28, 29].

The major strengths of our study are as follows: a large-scale, nationwide population-based study, inclusion of patients with acute stroke regardless of their age and hospitalization status, and appropriate age-standardization to WHO world population. Many previous stroke studies did not include older patients or only included hospitalized patients, thus excluding a proportion of patients and losing the full picture of stroke. Furthermore, we recruited patients who had undergone brain CT/MRI within 1 month of a new diagnosis of stroke. This reduced the possibility of coding error because almost all patients with acute stroke symptoms would be arranged to have brain imaging under the NHI program and stroke guidelines in Taiwan. Our study has some limitations. This was a retrospective cohort study, not a traditional “hot pursuit” study on stroke incidence through door-to-door interviews. Thus, we could not totally exclude the possibility of incomplete case ascertainment or a few coding errors. Nevertheless, the Taiwan NHIRD data have been validated, achieving a high accuracy rate of diagnosis of stroke [30]. In addition, data regarding IS and ICH subtypes, stroke severity scores, and some risk factors (e.g., smoking and alcohol intake) were not available in the NHIRD, which precluded us from doing relevant analyses. However, advanced developments of information technology, improvement of data collection in the stroke registries at primary healthcare centers, and centralized health insurance system would facilitate well-designed epidemiological studies in the future.

## Summary

In Taiwan, the incidence rate of first-ever stroke and the rate of early case fatality decreased significantly in both Chinese men and women in the early 21st century; men always had a higher incidence rate of stroke than women. Among pathological subtypes of stroke, the incidence of ICH decreased markedly in both sexes, while the incidence of IS decreased slightly; however, the decrease of IS incidence was significant in only women. Our study has shown the changes of stroke epidemiology and sex differences in Taiwan in the early 21st century, the potential targets to improve, and the need to develop effective strategies for stroke prevention at the individual and population levels.

## Supporting information

S1 Fig Inclusion of acute stroke patients. (DOCX)

S2 Fig Secular trends of intracerebral hemorrhage to ischemic stroke incidence ratio. (DOCX)
