# Supplementary material for: Incidence, subtypes, sex differences and trends of stroke in Taiwan
Source: PLoS One. 2022 Nov 16;17(11):e0277296. doi: 10.1371/journal.pone.0277296 (PMC9668115; doi:10.1371/journal.pone.0277296)
Supplement: S1 Fig — (DOCX) [file pone.0277296.s001.docx]

**S1 Fig. Inclusion of acute stroke patients.**

A National Health Insurance research database for a random sample of 2 million patients ever having brain or renal disease as a major diagnosis from 1997 to 2011

(N=2,000,000; the random sampling fraction=71%)

Including patients with a new diagnosis of acute stroke with pathological type classification of the following ICD-9 codes - subarachnoid hemorrhage (430), intracerebral hemorrhage (431), ischemic stroke (433.01,433.11, 433.21, 433.31, 433.81, 433.91, 434.01, 434.11, 434.91), and unclassified type (436) from 2004 to 2011

(N= 480,922)

Excluding patients without recent brain CT/MRI within one month of new diagnosis of stroke, with previous stroke, traumatic brain hemorrhage, subdural/epidural haemorrhage, brain tumor, or transient ischemic attack (N=109,008)

Incident patients of stroke with recent brain CT/MRI within one month of new diagnosis of stroke

(N=371,914)

Excluding missing data of sex and birth date

(N=68)

Incident patients of stroke with CT or MRI examinations from 2004 to 2011

(N= 371,846)
