# Supplementary material for: Incidence, subtypes, sex differences and trends of stroke in Taiwan
Source: PLoS One. 2022 Nov 16;17(11):e0277296. doi: 10.1371/journal.pone.0277296 (PMC9668115; doi:10.1371/journal.pone.0277296)
Supplement: S2 Fig — (DOCX) [file pone.0277296.s002.docx]

**S2 Fig. Secular trends of intracerebral hemorrhage to ischemic stroke incidence ratio.**

ICH=intracerebral hemorrhage; IS=ischemic stroke; P_A=_p for trend in all; P_M=_p for trend in men; P_W_=p for trend in women.
